# 3D Bioprintable Self-Healing Hyaluronic Acid Hydrogel with Cysteamine Grafting for Tissue Engineering

**DOI:** 10.3390/gels10120780

**Published:** 2024-11-28

**Authors:** Kasula Nagaraja, Amitava Bhattacharyya, Minsik Jung, Dajeong Kim, Mst Rita Khatun, Insup Noh

**Affiliations:** 1 Department of Chemical and Biomolecular Engineering, Seoul National University of Science and Technology, Seoul 01811, Republic of Korea; 2 Convergence Institute of Biomedical Engineering and Biomaterials, Seoul National University of Science and Technology, Seoul 01811, Republic of Korea; 3 Medical Electronics Research Center, Seoul National University of Science and Technology, Seoul 01811, Republic of Korea

**Keywords:** hyaluronic acid hydrogel, cysteamine, 2-hydroxyethyl acrylate, self-healing, 3D bioprinting

## Abstract

The abundance of hyaluronic acid (HA) in human tissues attracts its thorough research in tissue regenerating scaffolds and 3D bioprintable hydrogel preparation. Though methacrylation of HA can lead to photo-crosslinkable hydrogels, the catalyst has toxicity concerns, and the hydrogel is not suitable for creating stable complex 3D structures using extrusion 3D bioprinting. In this study, a dual crosslinking on methacrylated HA is introduced, using cysteamine-grafted HA and varying concentrations of 2-hydroxy ethyl acrylate. The resultant hydrogel is suitable for extrusion 3D printing (or bioprinting), mechanically robust, self-standing, stable in phosphate-buffered saline at 37 °C for more than 42 days, has high water absorption capacity with a low swelling ratio (1.5), and exhibits self-healing and adhesive properties. Complex 3D structures like ears and pyramid shapes with more than 2 cm of height are 3D printed using the optimized composition. All the synthesized hydrogels have shown nontoxicity and cell-supportiveness. Loading of cells, tetracycline, and bovine serum albumin into the hydrogel led to better bioink properties such as cell attachment, growth, and proliferation for osteoblast cells. The test results suggest that this hydrogel is biocompatible and has potential for 3D bioprinting of self-standing structures in bioink form in tissue engineering and regenerative medicine.

## 1. Introduction

Hyaluronic acid (HA), a naturally occurring non-sulphated glycosaminoglycan (polysaccharides), is a major component in the extracellular matrix (ECM). It is found in all connectives, epithelial, and neural tissues. It has critical roles in tissue engineering, such as cell adhesion, proliferation, migration, and differentiation [1]. Due to these biological properties, it is widely researched by many groups for various tissue engineering applications. When formulated into a hydrogel, HA can provide a supportive 3D scaffold conducive to tissue regeneration by modulating diverse conditions, such as crosslinking methods and crosslinking agents [2]. HA-based hydrogels are used in several biomedical applications, including injectable hydrogels and 3D-printable scaffolds for tissue engineering, wound dressing, and drug delivery. As a biomaterial in tissue engineering, it has excellent biocompatibility, biodegradability with nontoxic byproducts, and the property to mimic the ECM [3]. The properties of HA hydrogel, such as its high water content and viscoelastic nature, make it ideal for not only encapsulating cells and bioactive molecules but also facilitating drug delivery and cell therapy to the target disease sites [4]. Even though hyaluronic acid bioink showed excellent properties in its natural form with different molecular weights and entanglements, it still faces several challenges. These include issues like printability, control of self-healing properties, bioink formation with cell and drug loading, bioprintability with cell loading and post-printing fidelity, and mechanical properties for tissue engineering applications, necessitating further physicochemical modifications and fabrication methods.

Several researchers have successfully utilized HA hydrogels for regenerating skin, bone, cartilage, and neural tissues. During cartilage repair using HA hydrogel, it improves tissue integration and function by promoting chondrocyte proliferation and ECM production [5]. Furthermore, its immune-modulatory properties support reduction of inflammation and scarring degrees, which is crucial for effective tissue regeneration [6]. However, physically crosslinked HA hydrogel has difficulty controlling long-term in vivo stability, including its mechanical strength. Hence, chemical modifications such as methacrylation and thiolation to the HA network are preferred to achieve stable hydrogels in tissue engineering applications [7].

Biocompatible crosslinking mechanisms have been researched by researchers for the gel formation of HA [8]. Among them, HA-methacrylate (HAMA) hydrogel is studied in tissue engineering and regenerative medicine for control of its physical properties and biocompatibility [9]. The chemically stable HAMA hydrogel, formed by UV light in the presence of a photo initiator [10], has high water retention similar to ECM properties, which supports cell viability and proliferation. In addition, growth factors, drugs, or nanoparticles can be loaded into the HAMA hydrogels during their gel formation [11]. Though it has numerous advantages, as stated above, the photocatalyst has toxicity concerns in tissue engineering applications, and the HAMA hydrogel may not be suitable for creating stable, complex, and large 3D structures.

HA-cysteamine (HACY) hydrogel is a novel biomaterial that combines the HA with the bioactive functionalities of cysteamine, a non-hormonal physiological regulator with both antioxidant and stress-relief activities. This makes HACY highly suitable for compression-related tissue engineering applications [12]. By grafting cysteamine, the modified HACY hydrogel is expected to enhance redox-responsive characteristics, ensuring that it can be oxidized to release during the tissue regeneration process, thus not only minimizing the risk of chronic inflammation but also facilitating tissue integration [13]. These functions will be helpful for cartilage regeneration and healing. HACY hydrogels are expected to be particularly effective in regenerative medicine due to their enhanced bioactivity. The cysteamine component released from the hydrogel would act as a bioactive molecule that promotes cell adhesion and proliferation [14]. This makes HACY or thiolated HA hydrogels suitable for a wide range of applications, including cartilage repair, wound healing, drug delivery, and nerve regeneration in tissue engineering [15]. In appropriate concentrations, cysteamine-modified hydrogels can promote cell adhesion, proliferation, and differentiation [16]. 2-Hydroxyethyl acrylate (HEA) is an effective crosslinker for functional HA hydrogels through addition polymerization. With both hydroxyl and acrylate groups, HEA helps the formation of robust, covalently bonded networks, increasing the mechanical properties and stability of HA hydrogels [17]. When used as a crosslinker, HEA introduces acrylate groups into the HA backbone, enabling photopolymerization or chemical crosslinking to form a three-dimensional network of hydrogel. This results in hydrogels with adjustable mechanical properties, tailored to match the application-specific requirements of different tissue types such as cartilage and bone [18]. The mechanical strength provided by HEA crosslinking supports the structural integrity necessary for load-bearing tissues, while the hydrogel’s biocompatibility and biodegradability facilitate seamless tissue integration and regeneration. Moreover, the versatility of HA-HEA hydrogels allows easy incorporation of bioactive molecules, e.g., growth factors and drugs, enhancing the therapeutic efficacy of the scaffold. This makes HA-HEA hydrogels a promising platform for advancing regenerative medicine, providing customizable, robust, and bioactive scaffolds tailored to the needs of diverse tissue engineering applications. Cell support of the self-crosslinking hydrogel is essential for the bioink in 3D bioprinting-based tissue engineering. The advantages are not only cysteamine and hydroxyethyl acrylate employment but also the self-crosslinking mechanism between cysteamine and acrylate groups utilized for creating a HA-based hydrogel suitable for extrusion 3D bioprinting.

To achieve our research goal, we have synthesized HAMA (hyaluronic acid methacrylate) and HACY (hyaluronic acid cysteamine) and crosslinked them in the presence of different concentrations of HEA (2-hydroxyethyl acrylate). Four different amounts of HEA were used to prepare the final crosslinked hydrogels. The amounts of each component taken for preparation of the hydrogel samples are given in Table 1, along with the sample codes. The gel properties were examined to find the composition suitable for extrusion-based 3D printing and bioprinting, as well as injectable hydrogel. Different 3D architectures were developed, and the biological properties of each were evaluated, looking into its potential application as a bioink matrix for bone and cartilage tissue engineering.

## 2. Results and Discussion

### 2.1. Functional Groups and Surface Morphology Analysis

**Figure 1:**
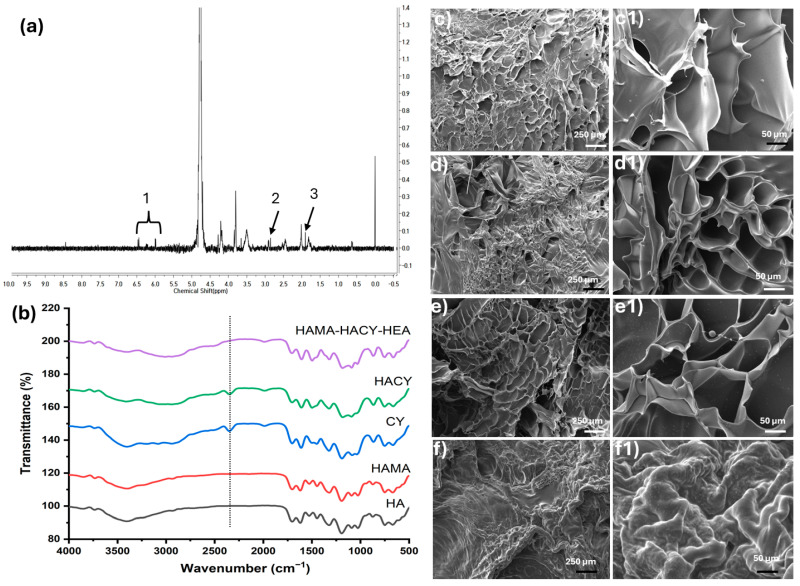
(**a**) NMR of 700 µL HEA hydrogel. (**b**) FTIR of raw materials, intermediate components and final hydrogel, and SEM analysis of four-different hydrogels. (**c**,**c1**) 300 µL HEA, (**d**,**d1**) 500 µL HEA, (**e**,**e1**) 700 µL HEA, and (**f**,**f1**) 1000 µL HEA.

Cysteamine-modified HA and methacrylated HA are dual-crosslinked with HEA in the presence of KPS (potassium persulfate). Figure 1a shows the NMR spectra of crosslinked hydrogel. Raw materials and intermediate products’ NMRs are given in Supplementary Figure S1. In NMR spectra, the zone shown as 1 represents two protons on methacrylate alkene [19]. Acrylate protons are also in region 1 [20]. The NMR peak marked as 2 is NH-SH cysteamine modification of hyaluronic acid. The peaks at 2.9 ppm and 3.04 ppm are due to the methylene protons of cysteamine as observed in the HA-SH spectrum [21]. The peaks marked as 3 represent the methyl group’s three protons of HA [19]. In FTIR, HA backbone peaks are strong and visible in all spectra (Figure 1b). The peak at 2340–2360 cm^−1^ is the cysteamine signature peak of the S–H bond (free H–S cysteamine) [22]. This peak is not visible in the final crosslinked product, indicating no free cysteamine in the crosslinked hydrogel. Cysteamine hydrochloride, used in modifying hydrogels for tissue engineering, has potential toxicity concerns. High concentrations can cause cytotoxic effects, necessitating careful dosage and thorough purification to ensure safety. Its biocompatibility largely depends on the formulation and application method [16]. Hence, ensuring the absence of free cysteamine is essential considering the hydrogel’s cell-supportive application.

The SEM analysis (Figure 1c–f) shows the surface morphology of the lyophilized hydrogels. The pores of 300 µL HEA are large, collapsed, and non-uniform. The uniform porous structures are observed for 500 µL HEA and 700 µL HEA samples. For 500 µL HEA samples, most of the pores are ~30–70 µm, while for 700 µL HEA samples, pore sizes are higher (~60–120 µm). These porous crosslinked structures help the permeation of oxygen and nutrients to the embedded cells. The porous structure is absent in 1000 µL HEA.

### 2.2. Mechanical Properties Analysis

**Figure 2:**
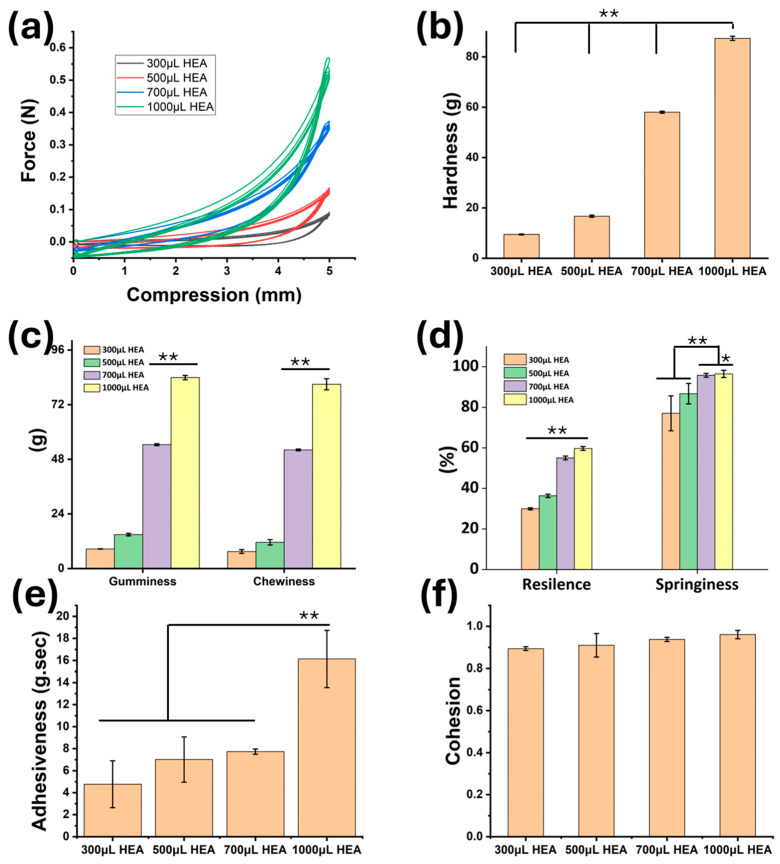
Mechanical texture properties of hydrogels: (**a**) cyclic compression (5 cycles), (**b**) hardness, (**c**) gumminess and chewiness, (**d**) resilience and springiness, (**e**) adhesiveness, (**f**) cohesion. (** *p*  ≤  0.01, * *p*  ≤  0.05).

The integration of HEA into the HA polymer matrix creates a scaffold with both high resiliency and adaptability. The cyclic mechanical properties of HAMA-HACY-HEA hydrogels improve significantly with the increase in the amount of HEA used to crosslink the hydrogels (Figure 2a). The five-cycle compressive strength curves with 50% compression show excellent recovery of all compositions as the compressive curves follow the initial trace path throughout the test. The 3D bioprintable hydrogel scaffolds are not as robust as the native tissue, or the permanent/semi-permanent implants used for cartilage repair. The hydrogel scaffolds are cell-included, and it is expected that the cells grow and proliferate to cover the entire 3D bioprinted scaffold and regenerate the tissue, mimicking the native one. Looking into these, the mechanical properties of a 3D bioprintable scaffold cannot be in the range of the native tissue. However, to maintain the structural integrity during the cell culture period, it should have adequate mechanical properties. Around 2 kPa yield stress is reported by the researchers using HA-based bioink for 3D bioprinting purposes [23]. Considering 10 mm diameter samples used for the compressive strength study, ~4.5 kPa compressive strength is observed at 50% compression for the 700 µL sample. Moreover, this is not the yield stress; hydrogel takes back its shape after the compressive cycle, so yield stress should be greater than these values. Not only does the strength improve, but also the hardness, chewiness (product of gumminess and springiness) and gumminess (product of hardness and cohesiveness) are significantly improved from 300 µL HEA to 1000 µL HEA (Figure 2b,c). Properties like resilience and springiness indicate structure retention ability after compressive cycles [24]. These properties are significantly better for 700 µL HEA and 1000 µL HEA samples as compared to 300 µL HEA and 500 µL HEA. The adhesiveness of the hydrogel to the test metal surface (aluminum) is significantly higher for 1000 µL HEA samples than the other samples. However, the cohesion between layers is not significantly different, indicating the suitability for printing using different compositions. These results suggest that it is a soft hydrogel with more than 90% springiness. The cyclic compressive force curve shows that the samples can withstand 50% cyclic compression without much hysteresis loss. Looking into the surface morphology and these mechanical texture properties, the 700 µL HEA sample shows greater promise to create multilayer, larger, and more complex structures with improved self-standing 3D printing ability for cartilage repair and soft tissue regeneration.

### 2.3. Physical Properties of Gels (Rheology, Degradation, Water Absorbency, and Swelling)

**Figure 3:**
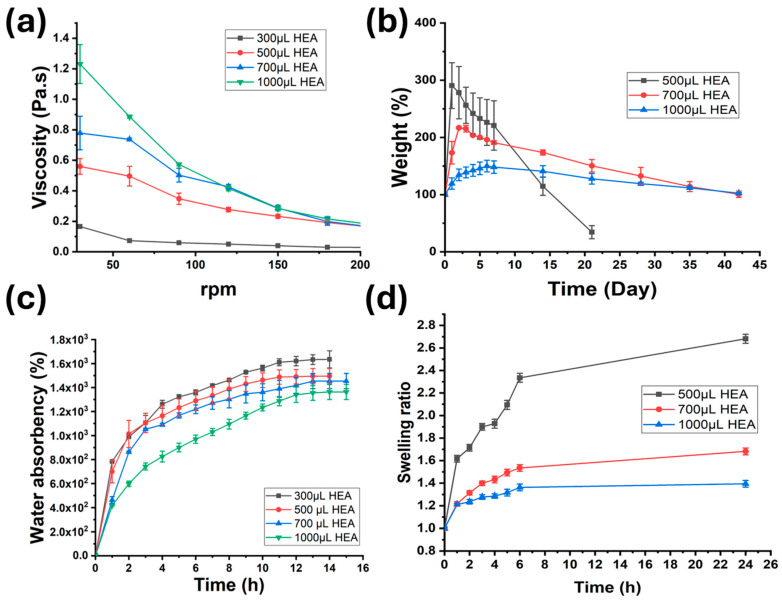
Physical properties of different compositions of hydrogels: (**a**) viscosity, (**b**) degradation, (**c**) water absorbency, (**d**) swelling ratio.

Figure 3 shows different physical properties of hydrogels synthesized in this study. The viscosity of hydrogels measured at different rpm indicates the shear thinning nature of the hydrogels (Figure 3a). While 300 µL HEA cannot withstand the high shear rate above 90 rpm and its viscosity falls to near zero, the other compositions have reasonable viscosity even at 240 rpm. This viscous property would help these compositions to extrude for 3D printing or bioprinting. The degradation in PBS at 37 °C (normal body temperature) shows stability in weight retention for 700 µL HEA and 1000 µL HEA samples up to 42 days, while the 300 µL HEA sample degrades or disintegrates within a day (shown in the Supplementary File) and 500 µL HEA shows sharp weight loss within 20 days. The initial weight gain is due to the highly water-absorbing nature of the hydrogel, which reduces with the increase in crosslinking density. The water absorbency of the lyophilized hydrogels shows very high-water absorption capacity for all the compositions. With an increase in HEA amount in the hydrogel, the water absorbency reduces to almost half compared to 300 µL HEA samples. Within 10 h, all the hydrogels’ water absorbency stabilizes. The equilibrium water absorption ratio or mass swelling ratio was used to calculate the crosslinking density of the hydrogels. The average values (in mol/cm^3^) for 300 µL HEA, 500 µL HEA, 700 µL HEA, and 1000 µL HEA samples are 1.50 × 10^−8^, 1.74 × 10^−8^, 1.83 × 10^−8^, and 2.03 × 10^−8^, respectively. All the compositions have shown less water absorbency (or the swelling ratio on a weight basis) than the reported values for hydrogel-based 3D bioprinting [25] and thus qualify for the application. More crosslinking means less swelling; however, that will compromise the extrusion ability. The diameter swelling mostly affects the post-printing structural fidelity. The extruded lines of 700 µL HEA and 1000 µL HEA samples were tested for their diameter swelling ratio after printing. The 500 µL HEA sample was found to keep swelling even after 24 h and swelled to more than 2.5 times its original diameter. Though 1000 μL HEA has the lowest swelling ratio (~1.3) due to higher crosslinking, it is hard to extrude and cannot be extruded smoothly using fine needles. Considering all these, the 700 μL HEA was selected for the drug loading and 3D bioprinting. The 700 μL HEA has a ~1.5 swelling ratio after 6 h, which is reasonably stable until the end of the study (24 h).

### 2.4. 3D Printability

**Figure 4:**
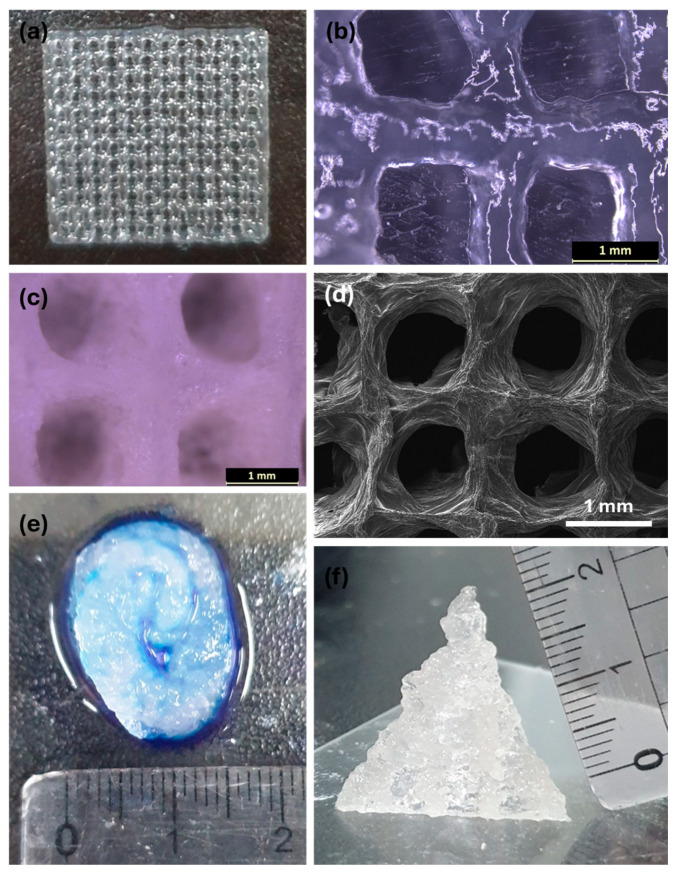
3D printing with 700 µL HEA hydrogel; 2 cm × 2 cm grid shape: (**a**) digital camera image, (**b**) microscope image, (**c**) microscope image after lyophilization, (**d**) SEM image, (**e**) ear shape, (**f**) pyramid shape.

Supplementary Figure S2 shows the 3D printing of all combinations to a multilayered hollow cylindrical structure. As indicated earlier, 1000 μL HEA is too hard to extrude and 3D print, and hence, not studied. Among other combinations, 700 μL HEA performs the best and retains its more than 1 cm height. The hydrogels 300 μL HEA and 500 μL HEA cannot retain their shape, and collapse. Hence, the complex and finer structures are 3D printed using 700 μL HEA crosslinker (Figure 4). A Supplementary Video File shows the high-resolution grid shape printing using this hydrogel. The extrusion ability and print resolution can be observed from the grid structure printed using this hydrogel (Figure 4a–d). A 432 ± 27 µm strut size and a distance between the struts of 1000 µm with a variation of 112 µm are maintained throughout the total 2 cm × 2 cm square grid-printed structure. The expected grid separation distance was selected as 1000 µm in the G-codes, and the 23G-A needle has a 337 µm inner diameter. Considering these values, the extrusion 3D printing ability of this hydrogel is excellent for multilayered complex structures. Further, structures like an ear shape (Figure 4e) and a more than 2 cm height pyramid shape (Figure 4f) were printed to observe the gel’s printability of complex structures. The shape retention and printing show the optimized hydrogel composition can create such structures with reasonable accuracy and self-standing ability.

### 2.5. Self-Healing and Adhesive Properties

**Figure 5:**
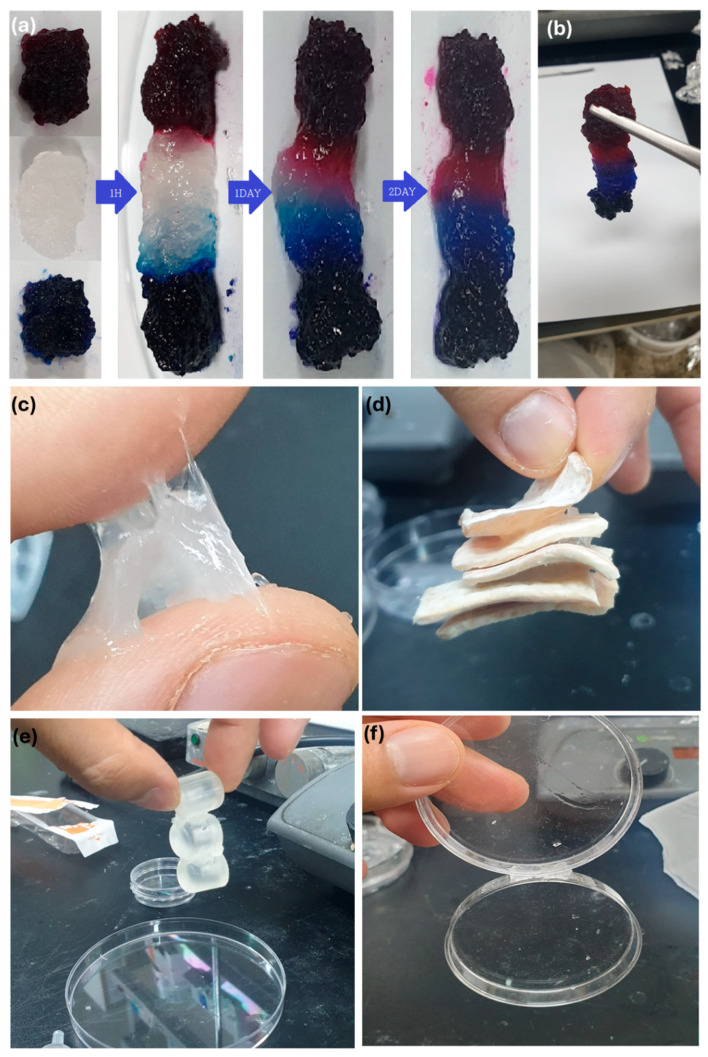
(**a**) Self-healing of 700 µL HEA hydrogel up to 2 days. (**b**) Self-healed 700 µL HEA hydrogel lifted using forceps. Adhesiveness of 700 µL HEA hydrogel between: (**c**) two fingers at separation more than 2 cm, (**d**) multiple pork skins, (**e**) resin tubes, (**f**) polystyrene plates.

Thiolated HA and HAMA have self-healing and adhesive properties in combination with other polymers [26,27]. Hence, the 700 µL HEA hydrogel sample was tested for its self-healing and adhesiveness properties (Figure 5). When kept in touch with each other, the three pieces of hydrogel started joining, as can be observed from the diffusion of dye molecules within 1 h, and, after 48 h (2 days), the three pieces of the hydrogel joined completely (Figure 5a). When lifted with forceps and shaken (Figure 5b), it was found that the three pieces were joined firmly to each other and extrudable using a needle and syringe without causing any discontinuity. For the adhesiveness test of the hydrogel, while keeping in between index finger and thumb and trying to stretch it, it was observed that more than a 2 cm gap between the fingers cannot separate it from the fingers (Figure 5c). Other substrates like pork skin (Figure 5d), a resin tube (Figure 5e), and a polystyrene petri plate (Figure 5f) also show strong adhesiveness to this hydrogel.

### 2.6. In Vitro Drug/Biomolecule Release Study

**Figure 6:**
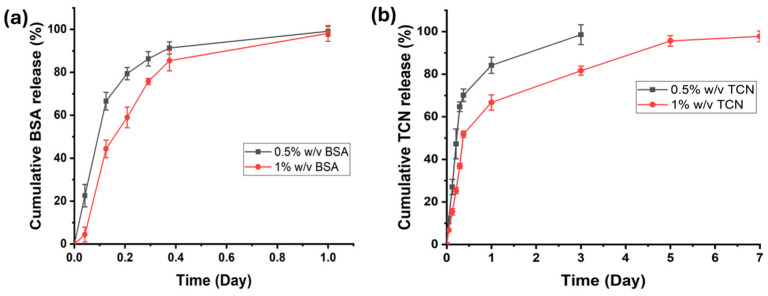
In vitro drug/biomolecule release study: (**a**) BSA, (**b**) TCN.

Tetracycline (TCN) is a commonly used antibiotic during cell culture experiments. This prevents bacterial growth and protects the cells. Usually, it is used in the cell culture medium. Similarly, bovine serum albumin (BSA) is a protein added to the cell culture medium to reduce oxidative cellular stress and damage. As cells are included in the hydrogel matrix, TCN and BSA loading inside the hydrogel should be advantageous for the cells. The standard calibration curves with known quantities of TCN and BSA were plotted from the UV-vis spectroscopy peaks (357 nm and 280 nm, respectively), as shown in Supplementary Figures S3 and S4. Figure 6 shows the cumulative release of TCN and BSA. While the total BSA is released from the hydrogel within a day, TCN takes 3 to 7 days to release from the hydrogel, almost completely (~99%). TCN, being a smaller molecule than BSA, is trapped inside the hydrogel more efficiently during the shear mixing within the twin-screw type extruder [28].

As almost all molecules were released, the Tanh function model is better suited to fit the data [29]. This model is derived from the Korsmeyer–Peppas model. We have used the following equation to explain the curves:

Qt = Qα × tanh(α × t^n^) (1)

Qα is the total amount released, α is a constant depending on size and diffusion constant of the drug. For Fickian diffusion, n = 0.45; for a non-Fickian diffusion mechanism, between 0.45 to 0.89, and n > 0.89 for Super Case II release from relaxed hydrogels. We have fitted our values in the model considering n = 1 and shown in Supplementary Figure S5.

### 2.7. In Vitro Cell Culture Studies

The potential toxicity and biocompatibility of 2-hydroxyethyl acrylate (HEA) are critical considerations for its use in tissue engineering. While HEA is effective as a crosslinker, it can pose cytotoxicity risks if residual monomers remain unreacted [30,31]. Careful purification and thorough polymerization processes are essential to minimize these risks. In this study, 48 h of dialysis was used with repeated changes of distilled water at every 12 h to remove any residual monomers. Biocompatibility studies have shown that, when properly crosslinked and purified, HEA-based hydrogels support cell viability and proliferation.

**Figure 7:**
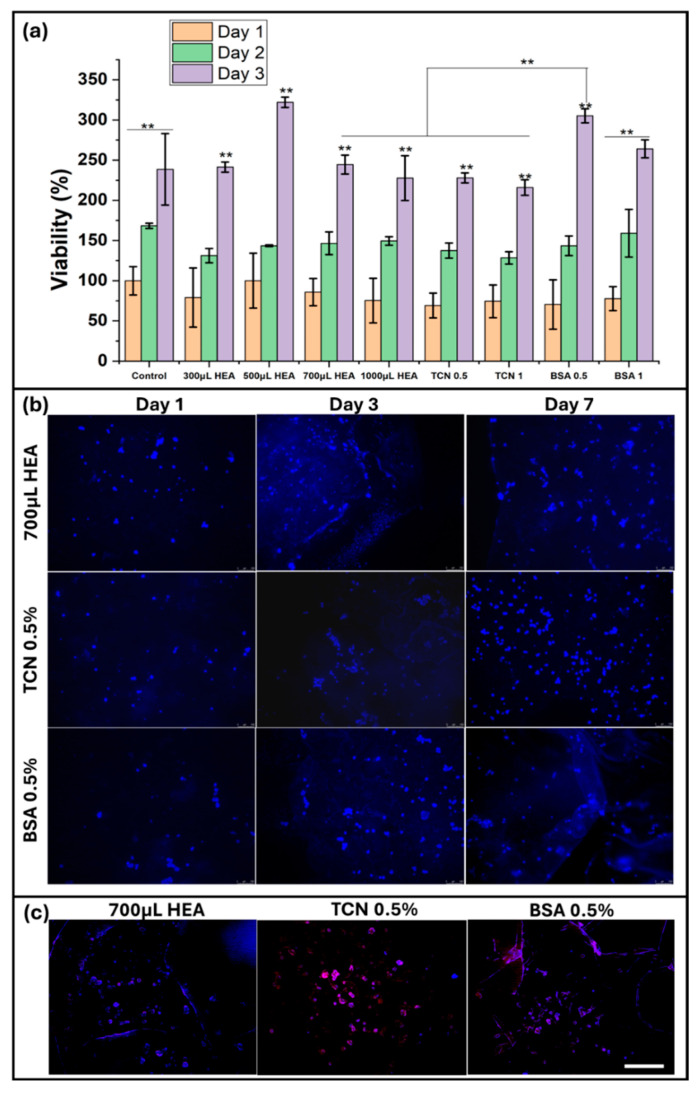
In vitro cell culture study for drug loaded and unloaded hydrogels: (**a**) cytotoxicity study (MTT assay)—cell viability up to day 3, (**b**) DAPI (nucleus—blue fluorescent) staining of cell embedded hydrogels at the end of Day 1, 3 and 7, (**c**) DAPI/phalloidin (nucleus/F-actin) merged image at the end of Day 7. (All images are in same magnification, Scale bar: 200 μm, ** *p* ≤ 0.01).

A cytotoxicity study of all hydrogel combinations and drug-loaded hydrogels was carried out using the MTT assay (Figure 7a). In the MTT assay, yellow tetrazolium salt is converted to crystals of purple formazan by dehydrogenase from the metabolically dynamic cells. The purple formazan amount corresponds to the quantity of viable cells. Consequently, this is utilized to survey the in vitro cytocompatibility and expansion in cell multiplication of proliferation of various hydrogels. For cytotoxicity evaluation through MTT assay, over 70% cell viability is viewed as nontoxic [32]. Cell viability estimated utilizing the MTT assay shows that every one of the gels is nontoxic and supportive to the cells. TCN and BSA were loaded with a 700 µL HEA sample, as it is the best among all combinations for extrusion 3D printing. However, among all HEA amounts used for crosslinking, 500 µL shows the best result in terms of viability. The cell proliferation is significantly higher for 500 µL HEA hydrogels. Among all drug-loaded samples, the 0.5% BSA-loaded 700 µL HEA hydrogel also exhibits significantly high cell growth on day 3. Use of 0.5% BSA improves the viability of 700 µL HEA samples significantly to the level of 500 µL HEA. This indicates that though the cell growth rate is affected by the use of a higher amount of HEA, the use of BSA can improve the cell viability significantly. Hence, the 3D printability and cell growth rate can both be taken care of.

The cell attachment, growth, and proliferation studies were conducted until day 7 with the extruded 700 µL HEA hydrogels (drug-loaded and unloaded). As more than 0.5% of drug loading did not offer much benefit (as observed in the MTT assay), 3D bioprinting of 700 µL HEA was carried out using 0.5% TCN and 0.5% BSA. The blue DAPI (nucleus staining) indicates uniform growth and proliferation of the MC3T3 cells within the gel matrix (Figure 7b). However, the day 7 DAPI/phalloidin (red) staining revealed that the cells attached to the BSA-loaded gel are spread and cell–cell communication established. The F-actin present in the cytoplasm is an indicator of cell health relevant to its growth and proliferation. The pure 700 µL HEA hydrogel has fewer actin filaments than TCN or BSA-loaded hydrogels. Hence, fully grown and highly proliferating cells can be achieved with these drug-loaded hydrogels. This can be useful for bone repair, as it promotes osteoblast activity and extracellular matrix production. The injectable form of this hydrogel may also facilitate minimally invasive surgical applications, making it a powerful tool in regenerative medicine for the small irregular defects that are very common in cartilage diseases.

## 3. Conclusions

Hyaluronic acid (HA) hydrogels offer a versatile and promising platform biomaterial for advancing 3D bioprinting and regenerative medicine, providing a bridge between biochemical research and clinical applications by utilizing the bio-physicochemical properties of the HA polymer itself—not only the inherent biocompatibility, biodegradation, and swelling properties but also the viscous, elastic, and non-immunogenicity properties. In this study, HA’s inherent bioactivity and mechanical properties have been further enhanced by chemical modifications of HA and drug/biomolecule loading into the self-crosslinking hydrogel, allowing for its tailored degradation rates. Furthermore, its modification enhanced mechanical properties to match both 3D bio-printing fabrication for soft tissue engineering scaffold and specific tissue engineering requirements. The versatility of HA hydrogel has been utilized and controlled, creating complex, multi-layered constructs that can better replicate the hierarchical structure of natural tissues through its chemical modifications. Furthermore, the addition of HACY into the HAMA hydrogels offers a versatile and responsive platform for tissue engineering through its chemical reaction of self-crosslinking, combining the benefits of HA with the unique properties of cysteamine to create a dynamic and biocompatible scaffold for regenerative medicine. The hydroxyl groups in HEA also contribute to the control of hydrophilicity, ensuring high water retention, which is critical for maintaining cell viability and controlling nutrient diffusion through the polymer network of the gel scaffold. The synthesis process produces a robust, three-dimensional scaffold capable of maintaining structural integrity under physiological conditions by controlling the ratio of functional groups in the polymer chains.

In addition to these physico-chemical and mechanical properties, the bioink formation with cell and drug loading properties of this HAMA-HACY-HEA self-healing hydrogel has significant potential across various tissue engineering applications, including cartilage repair, wound healing, and soft tissue regeneration. Furthermore, the bioprintability and mechanical properties of HAMA-HACY-HEA hydrogel are controlled by modulating the composition of HEA. This study is restricted to in vitro experiments, while more intense cell culture studies (in vivo and in vitro) are expected in the future to prove the potential of this hydrogel for cartilage repair, wound healing, and soft tissue regeneration. Overall, this hydrogel offers significant promise for advancing tissue engineering and improving patient outcomes.

## 4. Materials and Methods

### 4.1. Materials

Hyaluronic acid (HA, Mw = 1.659 MDa, PDI = 3.974) was kindly donated by Ildong Pharmaceutical company, Seoul, Republic of Korea (AK0701 batch). All other chemicals, including methacrylic anhydride (Mw: 154.16), cysteamine hydrochloride (Mw: 113.61), 2-hydroxyethyl acrylate (HEA, Mw: 116.12), 1-ethyl-3-(3-dimethylaminopropyl) carbodiimide (EDC, Mw: 155.24), N-hydroxy succinimide (NHS, Mw: 115.09), and potassium persulfate (KPS, Mw: 270.32), were purchased from Sigma Aldrich, Burlington, MA, USA.

The osteoblast cell line derived from mouse calvaria (MC3T3-E1, Young Science Inc., Bucheon, Republic of Korea), α-minimum essential media (MEM) (Sigma–Aldrich, Burlington, MA, USA), 10% fetal bovine serum (FBS, Gibco Korea, Seoul, Republic of Korea), penicillin-streptomycin (Sigma–Aldrich, Burlington, MA, USA), and LnD Max-view Live/Dead cell staining kit (Biomax, Guri, Republic of Korea) were used for cell culture studies.

### 4.2. Synthesis of Hydrogels

**Scheme 1:**
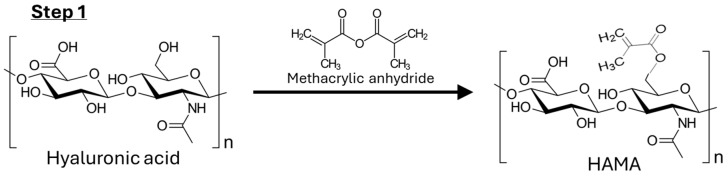
Synthesis reaction for HAMA.

**Scheme 2:**
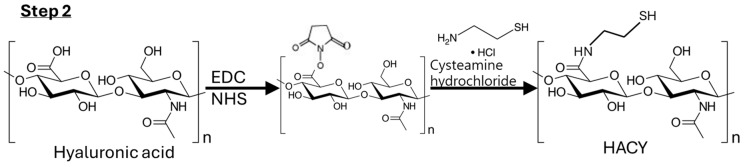
Synthesis reaction for HACY.

**Scheme 3:**
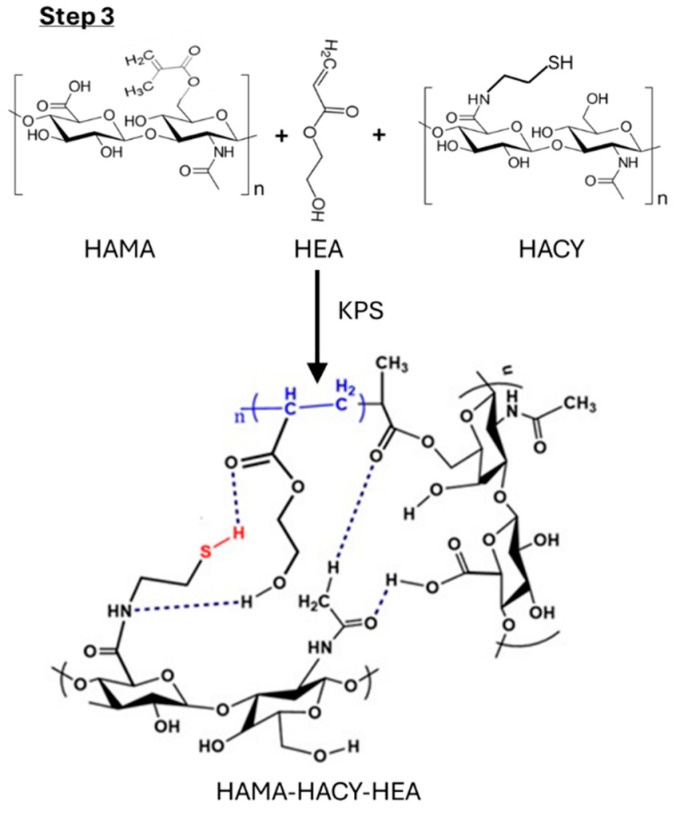
Synthesis reaction for HAMA-HACY-HEA hydrogel.

The synthesis and proposed reaction mechanisms are illustrated in Scheme 1, Scheme 2 and Scheme 3. The possible bonds are indicated in Scheme 3.

#### 4.2.1. Step 1: Synthesis of HAMA

Hyaluronic acid (1.0 g) was dissolved in distilled water (100 mL) in a 250 mL double-necked round-bottom flask by stirring overnight at room temperature. After dissolving, 7.5 mL of methacrylic anhydride was added dropwise and stirred for 24 h. pH was adjusted to 8.0–9.5 using a 3 mM sodium hydroxide solution. Unreacted methacrylate was removed by adding 30 mL of 70% ethanol to form a precipitate. After forming precipitation, excess ethanol was removed, and precipitations were collected. The product (Scheme 1) was dialyzed (Mw cut-off 6–8 kDa) with 5 L distilled water for 48 h with repeated water replacements at every 12 h, frozen at −80 °C for 24 h, and lyophilized for 5 days.

#### 4.2.2. Step 2: Synthesis of HACY

Hyaluronic acid (1.0 g) was dissolved in distilled water (100 mL) in a 250 mL two-necked round-bottom flask. Then, 1.2 g EDC and 1.6 g N-hydroxy succinimide were added to the HA solution. This mixture was allowed to react while stirring constantly for 5 h. After that, 1.8 g of cysteamine hydrochloride was added to the mixture and allowed to react overnight with continuous stirring. The product (Scheme 2) was dialyzed (Mw cut-off 6–8 kDa) with 5 L distilled water for 48 h with repeated water replacements at every 12 h, frozen at −80 °C for 24 h, and lyophilized for 5 days.

#### 4.2.3. Step 3: Preparation of HACY-HAMA-HEA Hydrogel

3% HACY solution and 3% HAMA solution were prepared. A quantity of 3 mL of HACY solution was mixed with 2 mL of HAMA solution and stirred for 3 h to get a homogeneous mixture. Furthermore, HEA was added in different concentrations. This reaction mixture was stirred for 3 h. Finally, 0.2 mL of 5% KPS was added, and the reaction solution was stirred for 30 min. This reaction mixture was allowed to stand for 50 min at 70 °C to form the final gel (Scheme 3). The final product, HAMA-HACY-HEA hydrogel (Scheme 3) was dialyzed (Mw cut-off 6–8 kDa) into 5 L distilled water for 48 h, with repeated water replacements at every 12 h.

### 4.3. Characterization

#### 4.3.1. NMR Analysis

A 700 MHz nuclear magnetic resonance (NMR) detector (model: DD2 700, Agilent Technologies, Santa Clara, CA, USA) was used to find the ^1^H-NMR spectra of the raw materials and hydrogel samples (after lyophilization of 5 days) in deuterium oxide (D_2_O) solvent.

#### 4.3.2. FTIR Analysis

For functional group analysis of the 5-day lyophilized gel samples and raw materials, attenuated total reflectance Fourier transform infrared spectroscopy (ATR-FTIR) was carried out with a spectrometer (Varian 640-IR, ALT, East Lyme, CT, USA), in the 400–4000 cm^−1^ wavelength range in powder form.

#### 4.3.3. Scanning Electron Microscopic Analysis

The surface morphologies of the four different composition gels were analyzed by SEM (Model: TESCAN VEGA3, Kohoutovice, Czech Republic). All samples for SEM were frozen at −75 °C for 24 h, lyophilized for 5 days, and then coated with platinum for 90 s with a sputter coater. All images were analyzed using ImageJ software (Version: 1.54d).

#### 4.3.4. Mechanical Properties

The mechanical texture properties of all four composition gels were measured using a Stable Micro System (TA. XT plus texture analyzer, Surrey, UK). The four composition synthesized gels were molded into cylindrical shapes (10 mm × 10 mm) and kept in the refrigerator for 24 h. For the cyclic compression test, 50% was given in the distance mode (with a maximum distance of 5 mm and test speed: 2 mm. s^−1^, 5 cycles). The data for 3D printing-related mechanical properties like hardness, gumminess, chewiness, resiliency, springiness, adhesiveness, and cohesion [24] were collected using the same machine.

#### 4.3.5. Rheology Analysis

The viscosity of all the hydrogels were analyzed using Brookfield viscometer, AMETEC, Inc., Berwyn, PA, USA (20 mm diameter, parallel plates with 1 mm spacing) at room temperature (25 °C). The test condition was 30 rpm to 240 rpm at an interval of 30 rpm, each data point has 10 measurements taken at every 1 s.

#### 4.3.6. Degradation Test

The in vitro degradation test of different hydrogels was evaluated by immersing 1 g of each gel in PBS at pH 7.4 and 37 °C after the standard synthesis process. The sample 300 μL HEA disintegrated within 1 day, and hence was found not suitable for the test and discarded. At regular intervals up to 42 days, the weight of the samples was measured. The degradation profile was obtained by measuring their percentage weight retained compared to day 0.

Weight retained (%) = (final wt./initial wt.) × 100(2)

#### 4.3.7. Water Absorbency Test

A quantity of 0.037 ± 0.003 g lyophilized (5 days of lyophilization) gel samples (each of the four compositions) were immersed into 10 mL PBS, pH 7.4, in a 12-well plate. At regular intervals, the gel samples were collected, and weights were measured. The percentage water absorbency was calculated using the following equation:

Absorbent capacity (%) = (wt. of wet sample − wt. of dry sample)/wt. of dry sample) × 100 (3)

The Flory–Rehner equation was used to relate the water absorbency (mass swelling ratio) and crosslink density.

Crosslink density: Ʋ = − (ln(1 − V_2_) + V_2_ + χV_2_^2^)/(V_1_(V_2_^1/3^ − V_2_/2)) (4)

Here,

V_1_ ~ 18.015 cm^3^/mol for water V_2_ = 1/(1 + Q) (5)

Q = Ws/Wd(6)

Ws = equilibrium swelled weight after water absorption; Wd = dry weight of the polymer; χ (Flory–Huggins interaction parameter) for water and hyaluronic acid is typically 0.439 [33].

#### 4.3.8. Swelling Test

The swelling study was done with the hydrogels after synthesis. The samples 500 μL HEA, 700 μL HEA, and 1000 μL HEA can be extruded and had reasonable stability, So, these gels were extruded using a 14-gauge needle and kept in PBS (pH 7.4) at 37 °C. At regular intervals (1, 2, 3, 4, 5, 6, 24 h), the diameter of the extruded gel lines was measured at 5 different places as marked in the samples, and the percentage swelling of the extruded lines was calculated using Equation (3).

Swelling (%) = (Final diameter—initial diameter)/Initial diameter) × 100 (7)

#### 4.3.9. 3D Printing

To test the 3D printability, all compositions of the prepared hydrogels were 3D printed into multi layered hollow cylinder using a 23G-A needle and 100 kPa extrusion pressure in a customized 3D bioprinter, as described previously [32]. The hydrogel 1000 μL HEA is too hard to extrude, as it is more crosslinked. Among the other compositions, 700 μL HEA was found to be best for extrusion, so it was used to print various shapes and used for further studies. SolidWorks (Dassault Systemes SolidWorks Corp, Waltham, MA, USA) was used to create 3D complex structures, and Simplify 3D version 4.0, USA was used to generate the G-codes.

#### 4.3.10. Self-Healing and Adhesive Property Test of the Optimized Gel

To demonstrate the self-healing property of 700 μL HEA hydrogel, the gel was split into three parts, one colored with red and another blue. Three parts were kept side by side attached in a peri plate and checked at regular intervals. To observe the adhesiveness property of hydrogel, it was studied for its ability to attach to various substrates like human skin, pork skin, a resin tube, and a polystyrene plate.

#### 4.3.11. In Vitro Drug/Biomolecule Release Study

Quantities of 1% (20 mg) and 0.5% (10 mg) *w*/*v* of tetracycline (TCN) and bovine serum albumin (BSA) were loaded into 2 mL gel (700 µL HEA) using Biopen (Matrix CellBio, Seoul, Republic of Korea) [28], and the drug-loaded hydrogels were put into lyophilizer for 72 h. After lyophilization, the in vitro drug release study was conducted at pH 7.4 and 37 °C in PBS buffer media. At regular intervals, the aliquot was collected, and the UV vis spectrum was taken. The cumulative drug release was calculated according to a standard drug solution.

#### 4.3.12. In Vitro Cell Culture Study

Cytotoxicity was studied using 3-(4,5-dimethylthiazol-2-y1)-2,5-diphenyl tetrazolium bromide (MTT) assay in an extraction protocol, as described previously [32].

The MC3T3 cells were seeded onto the 96-well plate with 500 cells/well (100 μL medium/well). The samples were assessed for cell viability on days 1, 2, and 3 using MTT assay.

For the in vitro cell culture studies, the hydrogels were sterilized under UV radiation for 1 day and repeatedly washed with both phosphate-buffered saline (PBS) and medium. A quantity of 0.5 million/mL MC3T3 cells (passage 14) was mixed with each hydrogel and bioprinted. Samples were stained with 4’,6-diamidino-2-phenylindole (DAPI) on days 1, 3, and 7 for the nucleus (blue). Rhodamine phalloidin was used for F-actin staining (red). The fluorescent images were captured by employing the filters in the fluorescence microscope (Leica DMLB, Wetzlar, Germany) and then merged using the LAS-X Leica microsystems software (Version: 3.3.0.16799).

### 4.4. Statistical Analysis

Other than cell culture studies, all characterization tests were carried out with five samples (n = 5). Cell culture studies were carried out in triplicate. All images were analyzed by employing ImageJ software (Version: 1.54d, NIH, USA) to get the information on pore sizes and live/dead or cellular behaviors on the gel surface. Statistical analysis was done by one-way analysis of variance with both 95% confidence (* *p* ≤ 0.05) and 99% confidence (** *p* ≤ 0.01) by employing Tukey’s Means Comparison Test with Origin 9.0 software.

## Figures and Tables

**Table 1:** Hydrogel sample codes and amount taken of each component during synthesis.

SI No.	Sample Code	HACY (*w*/*v*)	HAMA (*w*/*v*)	2-HEA	KPS (5%, *w*/*v*)
1	300 μL HEA	3% 3.0 mL	3% 2.0 mL	300 μL	500 μL
2	500 μL HEA	3% 3.0 mL	3% 2.0 mL	500 μL	500 μL
3	700 μL HEA	3% 3.0 mL	3% 2.0 mL	700 μL	500 μL
4	1000 μL HEA	3% 3.0 mL	3% 2.0 mL	1000 μL	500 μL

Note: Generalized code HAMA-HACY-HEA is used in some cases for the best composition 700 μL HEA.

## Data Availability

Data will be available from the corresponding author on request.

## Supplementary Materials

The following supporting information can be downloaded at: https://www.mdpi.com/article/10.3390/gels10120780/s1, Figure S1: NMR analysis of raw materials and hydrogel. Figure S2: 3D printed hollow cylinders with different hydrogels: (a), (b) 300 μL HEA front and top view; (c), (d) 500 μL HEA front and top view; (e), (f) 700 μL HEA front and top view. Figure S3: UV spectrum of different conc. Tetracycline (TCN). Figure S4: UV-spectrum of different conc. Bovine serum albumin (BSA). Figure S5: Proposed curve fittings of BSA and TCN in tanh model. Video S1: 3D bioprinting of self-healing hyaluronic acid hydrogel with cysteamine grafting
