# Supplementary material for: 3D Bioprintable Self-Healing Hyaluronic Acid Hydrogel with Cysteamine Grafting for Tissue Engineering
Source: Gels. 2024 Nov 28;10(12):780. doi: 10.3390/gels10120780 (PMC11675811; doi:10.3390/gels10120780)
Supplement: Supplementary file 1 [file gels-10-00780-s001.zip › gels-3307228-supplementary-v2/gels-3307228-supplementary-v2.pdf]

3D bioprintable self-healing hyaluronic acid hydrogel with cysteamine grafting for tissue engineering

Kasula Nagaraja[1+], Amitava Bhattacharyya[1,2,3+], Minsik Jung[1], DaJeong Kim[1], Mst Rita Khatun[1], Insup Noh[1,2*]

[1]Department of Chemical and Biomolecular Engineering, Seoul National University of Science and Technology, Seoul 01811, Republic of Korea

[2]Convergence Institute of Biomedical Engineering and Biomaterials, Seoul National University of Science and Technology, Seoul 01811, Republic of Korea

[3]Medical Electronics Research Center, Seoul National University of Science and Technology, Seoul 01811, Republic of Korea

*Email: insup@seoultech.ac.kr

3.6 NMR analysis:

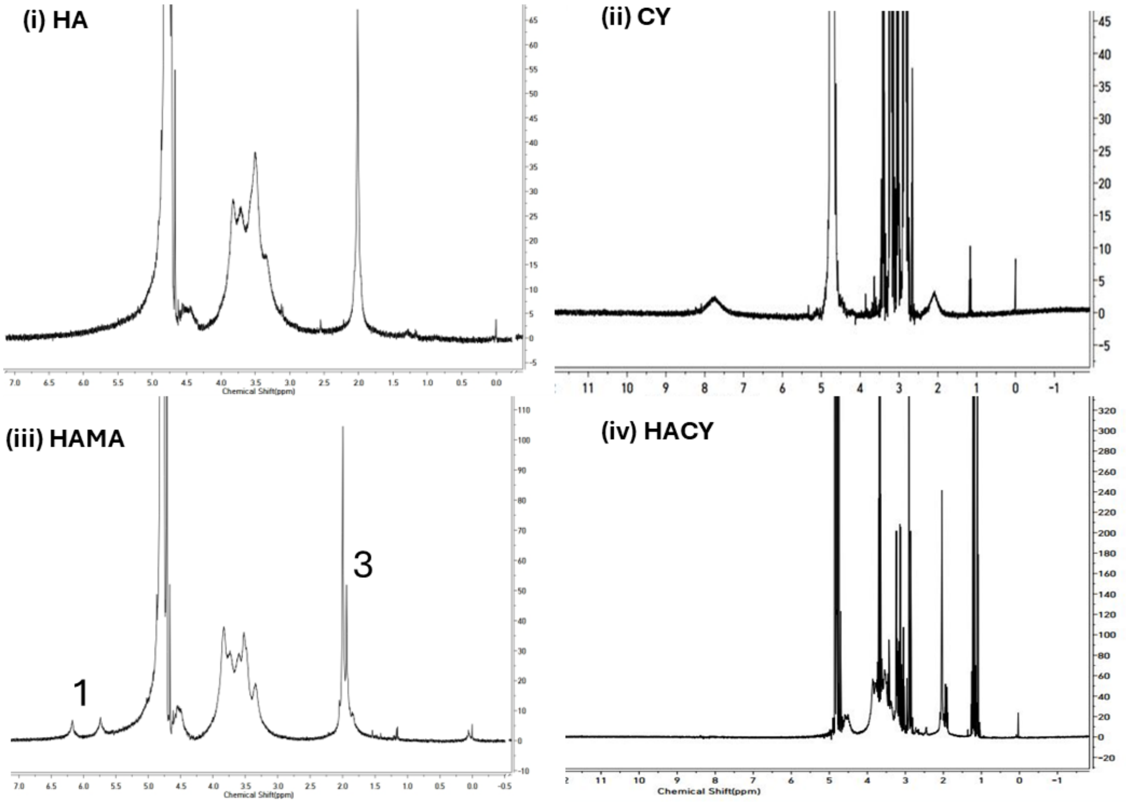

Figure S1. NMR analysis of raw materials and hydrogel.

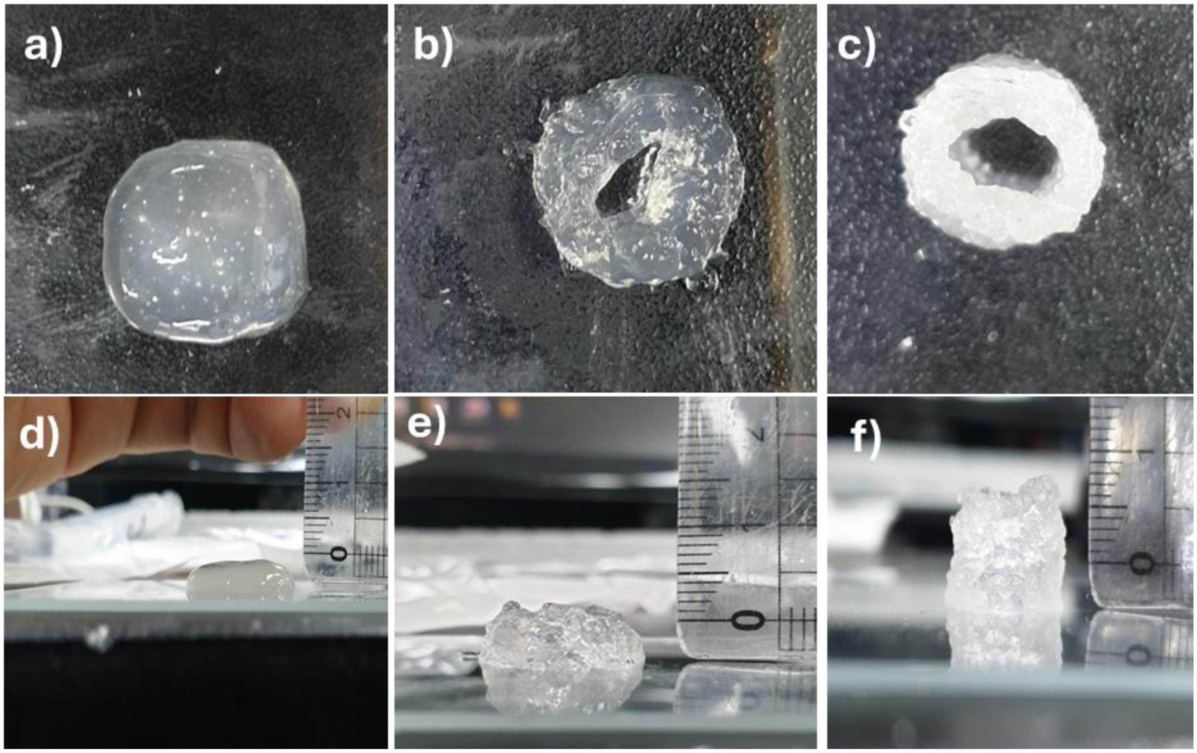

Figure S2. 3D printed hollow cylinders with different hydrogels: a), b) 300 HEA front and top view; c), d) 500 HEA front and top view; e), f) 700 HEA front and top view

Standard calibration equation for tetracycline release study:

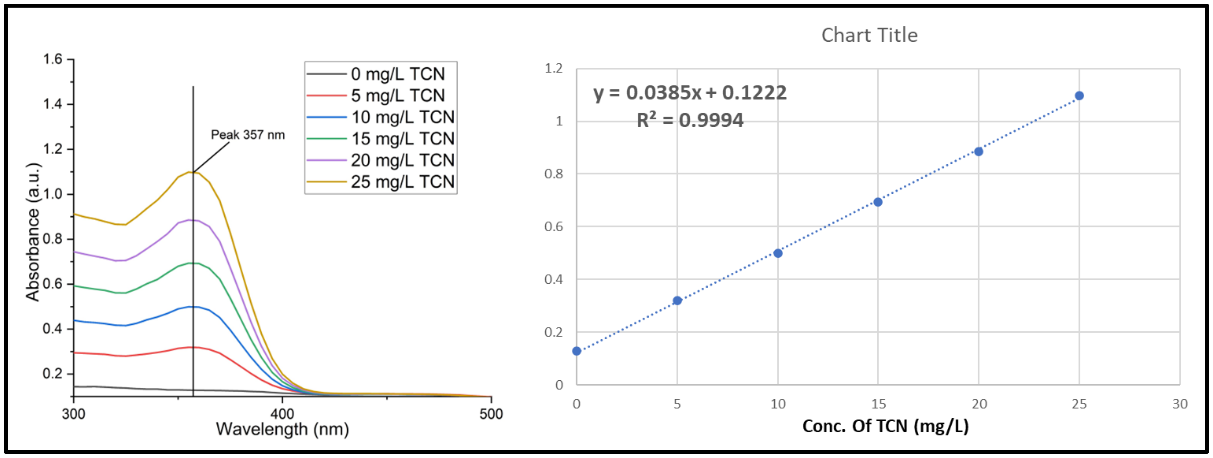

Figure S3. UV spectrum of different conc. Tetracycline (TCN).

Standard calibration equation for BSA release study:

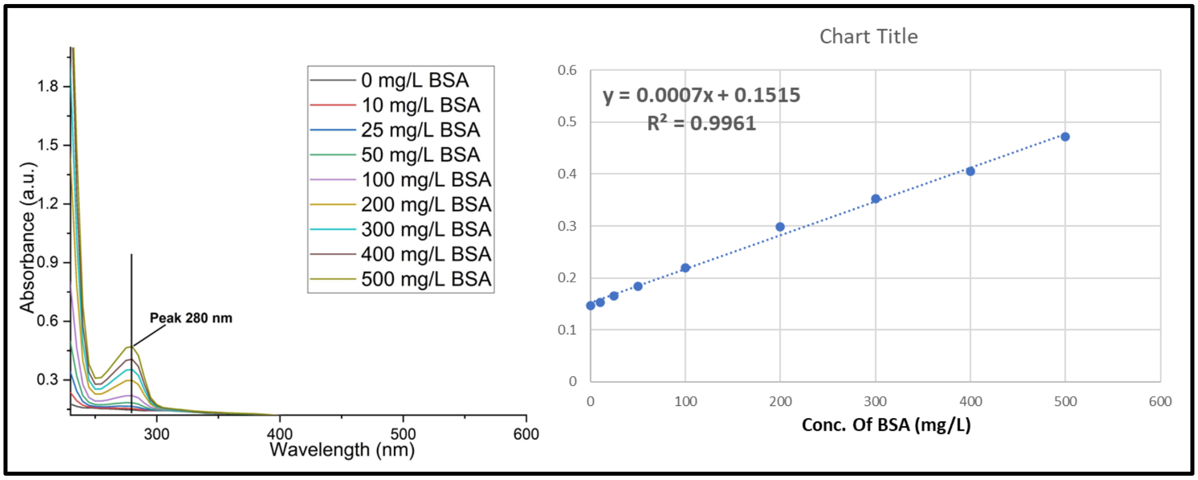

Figure S4. UV-spectrum of different conc. Bovine serum albumin (BSA)

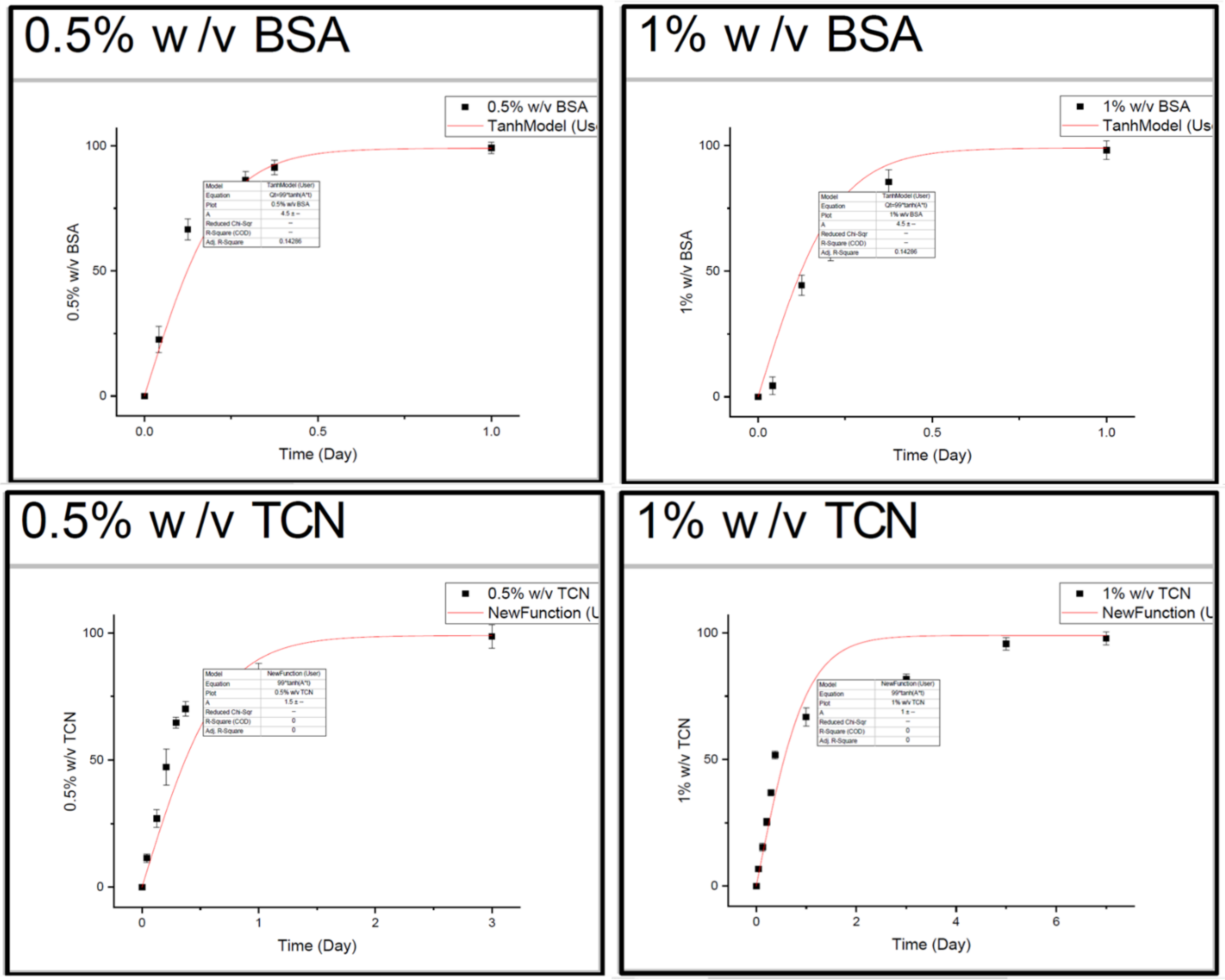

Figure S5. Proposed curve fittings of BSA and TCN in tanh model
